# Home Monitoring Delivered Through the Emergency Department for Outpatients With COVID-19: COVID19@Home Aachen Pilot Cohort Study

**DOI:** 10.2196/58364

**Published:** 2025-09-18

**Authors:** Lukas Niekrenz, Christian Hübel, Christopher Plata, Henning Biermann, Claas Leber, Lisa Sophie Schütze, Svea Holtz, Susanne Maria Köhler, Kim Deutsch, Nurlan Dauletbayev, Sebastian Kuhn, Beate Sigrid Müller, Christian Cornelissen, Rembert Koczulla, Gernot Rohde, Claus Franz Vogelmeier, Jörg Christian Brokmann, Michael Dreher

**Affiliations:** 1 Department of Pneumology and Intensive Care Medicine, University Hospital Aachen, RWTH Aachen University, Pauwelsstrasse 30, Aachen, 52074, Germany, 49 241800; 2 Department for Acute and Emergency Medicine, University Hospital Aachen, RWTH Aachen University, Aachen, Germany; 3 Institute of General Practice, Goethe-University Frankfurt, Frankfurt am Main, Germany; 4 Institute for Digital Medicine, University Hospital Gießen-Marburg, Philipps University of Marburg, Marburg, Germany; 5 Department of Internal, Respiratory and Critical Care Medicine, Philipps University of Marburg, German Center for Lung Research (DZL), Marburg, Germany; 6 Department of Pediatrics, McGill University, Montreal, Quebec, Canada; 7 The Research Institute of McGill University Health Centre, Montreal, Quebec, Canada; 8 al-Farabi Kazakh National University, Almaty, Kazakhstan; 9 Institute of General Practice, University of Cologne, Cologne, Germany; 10 Schön Klinik Berchtesgadener Land, PMU Salzburg, German Center for Lung Research (DZL), Marburg, Germany; 11 Department of Respiratory, Intensive Care and Sleep Medicine, University Hospital Gießen-Marburg, Philipps University of Marburg, Marburg, Germany

**Keywords:** home monitoring, telemonitoring, patient-led monitoring, nonsupervised home monitoring, physician-patient relationship

## Abstract

**Background:**

The overwhelming COVID-19 situation in 2020/2021 required novel approaches that did not require additional personnel within the current health care system. Therefore, we initiated a trial of nonsupervised home monitoring via the emergency department of a tertiary hospital without the support of a virtual ward as part of the “Netzwerk Universitaetsmedizin” cooperation in Germany. Given that daily vital sign checks for inpatients with COVID-19 could indicate clinical deterioration, this approach might also be helpful in an outpatient setting and could help to identify the need for hospitalization and additional resources.

**Objective:**

This study aims to determine whether patient-led home monitoring for acute SARS-CoV-2 infection can be implemented through the emergency department of a tertiary care provider.

**Methods:**

Patients who tested positive for SARS-CoV-2 infection in our emergency department between May 2021 and May 2022, did not have a medical indication for hospitalization, and were discharged to the outpatient setting were offered the opportunity to perform nonsupervised home monitoring of vital signs. Those who agreed to participate received Bluetooth-enabled devices to measure temperature, oxygen saturation, and blood pressure and downloaded a smartphone app. Participants were encouraged to measure their vital signs for at least 28 days. There was no virtual ward or real-time surveillance of the recorded data, but these could be made available to primary care physicians. Compliance with self-measurements was determined, and participants were contacted after the monitoring period for a semistructured interview.

**Results:**

A total of 828 patients with COVID-19 were treated at the emergency department. Of these, 262 were directly discharged into ambulatory isolation after initial assessment, 25 were offered the opportunity of nonsupervised home monitoring, 15 successfully activated the devices, and 9 performed more than one complete measurement using the app. These 9 participants used the devices for an average of 15.8 days after discharge. Interviewed participants reported various difficulties with device setup but said they were pleased to use home monitoring and felt that the measurement option gave them additional security.

**Conclusions:**

This study highlighted the challenges associated with implementing nonsupervised home monitoring for outpatients with COVID-19 who presented to the emergency department of a tertiary hospital. Implementing such a system without the involvement of additional personnel does not appear to be the optimal approach. We suggest that the physician-patient relationship might be a factor that is essential for the success of patient-led approaches to home monitoring.

## Introduction

During the COVID-19 pandemic, intensive efforts were made to find appropriate options for providing adequate care without overburdening the health care system. Given the number of severe acute respiratory distress syndrome cases and overcrowded intensive care units, medical staff were in short supply, especially in Germany [1-3]. The novel nature of COVID-19 and initial lack of knowledge about when and to what extent medical care and hospital admission were necessary caused uncertainty in the general population, which could have contributed to increased use of medical care for respiratory symptoms [4]. It also resulted in excessive demands on health care resources, such as those described for disaster situations [5]. Subsequent evaluations in Germany suggested that the majority of patients with COVID-19 could be treated on an outpatient basis [6].

The idea of using home monitoring for pandemic management arose from the fact that the regular vital sign checks performed on inpatients with COVID-19 could provide useful prognostic information. Such values were not routinely available for outpatients but could allow better assessment of these individuals and potentially provide an early signal of clinical deteriorations. This approach was implemented across many countries [7]. Home monitoring using eHealth and mobile health technologies provides data on relevant parameters for individual patients and helps with patient stratification [8].

Various home monitoring approaches include so-called virtual wards, that is, medical monitoring of the recorded data and contact by a treatment team [7,9-11]. In the context of COVID-19, contact with the medical team at all times has been reported to be a success factor for home monitoring [12]. In contrast, patient-led approaches without a virtual ward have been successfully used for monitoring chronic lung diseases (eg, the IPF online or I-FILE app) (Curavista bv) [13,14], but to our knowledge, they have not been used in the acute setting for infectious diseases.

At our institution during the height of the pandemic, our focus was on evaluating approaches that were feasible with existing (personnel) resources. With an already difficult staffing situation in the German health care system, regardless of the pandemic [15,16], we consider it essential that novel solutions require as few additional staff as possible. For this reason, we decided not to implement a virtual ward or real-time monitoring, but to establish patient-led home monitoring to supplement existing health care structures without providing personnel-intensive support. Therefore, as part of the egePan Unimed project for app-based telemedical home monitoring of COVID-19 [17], we focused on home monitoring as a possible approach to effectively managing outpatients with COVID-19.

This pilot study was designed to evaluate the feasibility by assessing patient usage data and medical use of an outpatient program of vital sign self-monitoring to detect early deterioration in patients with COVID-19 who presented to the emergency department but did not require hospital admission.

## Methods

### Study Design

This single-center cohort pilot study was conducted at the University Hospital Aachen, Germany (a large tertiary care provider) in accordance with STROBE (Strengthening the Reporting of Observational Studies in Epidemiology) reporting guidelines [18]. Recruitment was carried out by emergency department medical staff, supported by the study team for technical queries; no additional staff were made available to the emergency department to conduct the study.

### Participants

Eligible participants were adults who had tested positive for SARS-CoV-2 infection on a rapid antigen or polymerase chain reaction test and presented at the emergency department but did not have a medical indication for hospitalization and had access to their own smartphone. If a study participant was subsequently admitted to the hospital, their participation in the study was paused for the duration of hospitalization and could continue after discharge. Pregnancy was initially an exclusion criterion, but this was removed after re-evaluation of the risks of study participation for these individuals.

### Variables and Data Collection

Participants were given Bluetooth-enabled devices to record vital signs. Body temperature was measured using a noninvasive thermometer (Beurer FT 95 non-contact thermometer; Beurer GmbH), oxygen saturation (SpO2) was measured using a pulse oximeter (Beurer PO 60 pulse oximeter; Beurer GmbH), and blood pressure and heart rate were determined using a noninvasive sensor (Aponorm Basis Plus Bluetooth; WEPA Apothekenbedarf GmbH & Co KG). Participants were also given an installation code and emailed or given printed instructions on how to install the smartphone app (SaniQ Infect, Qurasoft GmbH). The study team contacted patients by telephone or video call to reaffirm that they met the inclusion criteria, to clarify remaining questions, and to provide support for device and app setup on the day after discharge from the emergency department.

Participants were told to measure their vital signs at least once a day for at least 28 days (or until symptoms resolved if these persisted for longer than 28 days). Measurements were designed to transmit from the measurement devices to the app via Bluetooth, but patients were also able to manually enter values into the app. During the self-monitoring period, any necessary treatment was provided by the appropriate primary care physician or the ambulatory health care system. Vital sign data collected by participants could be made available to their treatment providers (eg, primary care physicians) via PDF export.

Compliance with daily vital sign measurements was calculated as (number of actual measurements)/(number of target measurements). In addition, to account for days when measurements may have been taken more than once a day, daily adjusted compliance was calculated as the ratio of the binary variable (daily target of measurements reached or exceeded)/(number of days). The duration of vital sign self-measurement was also recorded.

After the end of the study, participants underwent a semistructured interview to gain insights about their experience with home monitoring. The semistructured interview format was developed within the research team. A translation of the original German interview guide can be found in Multimedia Appendix 1.

### Sample Size

Two hundred sets of monitoring devices were available for the study. The actual sample size was determined by the number of patients who requested treatment via the emergency department who could be included with the available staff.

### Statistical Analysis

Due to the low inclusion rate, k-anonymity and l-variability were low, meaning that reidentification appeared possible with additional knowledge. Therefore, only aggregated data are reported. These are presented using descriptive statistics. For the same reason, interview responses are reported only in summarized form. Qualitative analysis of interview responses was planned to be performed by 2 independent researchers following the principles of qualitative content analysis by Mayring [19].

### Ethical Considerations

The study protocol was approved by the Ethics Committee at the RWTH Aachen Faculty of Medicine (reference EK 099/21) and registered prospectively at the German Clinical Trials Register (ID DRKS00025123). All participants provided informed consent before enrollment in the study and could withdraw their consent at any time.

All recorded data were stored in an encrypted format on each participant’s smartphone. Participants were able to share their data with the study center via a reference code; participants could terminate transmission of their data in the app. Data were deidentified for processing at the study center after the end of the study phase and stored exclusively on ISO27001-certified data centers in Germany. The servers used were subject to German data protection law.

For hygiene reasons, study participants were allowed to keep the home monitoring devices provided at the end of the study. No further compensation was granted for participation. The standard retail price of the devices provided amounted to around US $250 at the time of the study.

## Results

### Participants

**Figure 1.**
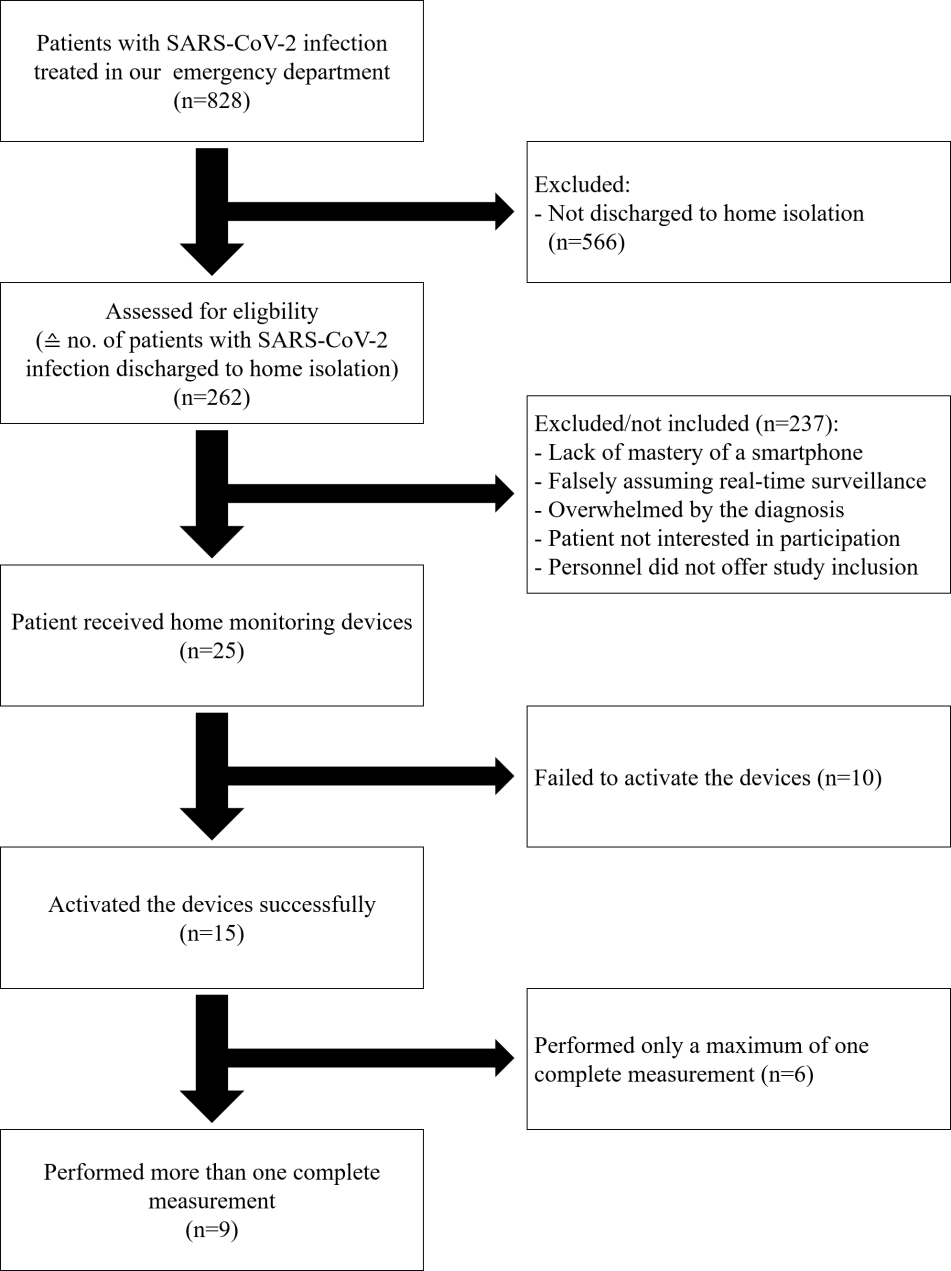
Study flowchart.

Between May 2021 and May 2022, a total of 828 patients with SARS-CoV-2 infection were seen in our emergency department, of whom 262 (31.6%) were discharged to home isolation after being assessed. However, the majority of these were not enrolled in the study. Reasons for nonenrollment were lack of mastery of a smartphone, falsely assuming real-time surveillance, being overwhelmed by the diagnosis of SARS-CoV-2 infection, not being interested in the study, or because health care professionals were overburdened and did not offer study participation (Figure 1). Of the 25 enrolled participants (median age 42 years, IQR 20 years; mean age 42.9 years, SD 14.3 years), 15 successfully activated the devices and app, and 9 of these (median age 39 years, IQR 25 years; mean age 44.4 years, SD 15.9 years) performed more than one measurement with each of the 3 devices using the app.

### Monitoring Usage and Compliance

The 9 participants who performed more than one complete measurement used the devices and app for an average of 15.8 days (range 1‐28 days). Compliance varied, with 4/9 active participants exceeding the absolute number of measurements, and 2 even exceeded the required measurements every day. Overall, there were 399 measurements out of a target of 426 measurements (93.7%). The required number of measurements was performed on 85/142 user days (59.9%). Overall compliance ranged from 22.2% to 200% (median 100%, IQR 58.4%; mean 102.5%, SD 56%). Daily-adjusted compliance ranged from 4% to 100% (median 81.8%, IQR 61.4%; mean 64.1%, SD 39.2%).

### Interview

Only 2 participants could be reached for the planned follow-up interview; an additional 4 agreed to a short informal conversation. For this reason, we decided to deviate from our planned approach to the analysis, as no meaningful evaluation seemed possible due to an insufficient number of participants. We decided to report the results only anecdotally. A formal analysis was not carried out. Nevertheless, in the setting of general practitioner care, an analysis was carried out based on a satisfactory number of cases, but this is reported elsewhere. The patients interviewed in our setting said that they were very pleased to use the home monitoring concept and felt that the measurement option provided additional security once the devices were set up. Various difficulties in setting up the devices were reported because this required each device to be paired with the patient’s smartphone. Performing measurements was considered to be time-consuming and required a willingness to deal with the devices. It was also reported that the time available for additional measurements without immediate feedback decreased after the end of the home isolation period. One participant reported that their device data led to further examinations being performed when they contacted their general practitioner.

## Discussion

### Principal Findings

This study highlighted significant challenges relating to the implementation of a home monitoring program by the emergency department of a tertiary care provider during the height of the COVID-19 pandemic.

Although many of the patients with SARS-CoV-2 infection treated in our emergency department were potentially eligible for home monitoring, the majority did not enter the trial. There were several reasons for this. First, a large number of potential participants were not offered information about the study because health care professionals were too busy to have the time to do this. Second, a relevant number of patients were excluded due to their lack of mastery of a smartphone (self-reported or assumed by personnel). This was often, although not exclusively, seen in older individuals. Fifty-five patients discharged to home isolation were aged 65 years or older, and only 3 of these consented to participate in the study. Third, it appeared that some of the features of the study intervention (self-directed monitoring, no additional physician-patient contacts, no virtual ward) created a perception of lack of security of follow-up, making consent less likely. Finally, many patients simply did not have any interest in participating in the study or were psychologically overwhelmed by being diagnosed with SARS-CoV-2 infection. In summary, apart from the issue of overburdened emergency room personnel, it was largely patient attitudes that probably led to the low inclusion rate.

In general, the emergency department of a tertiary care provider is unlikely to be the first port of call for many patients with viral infections, and this is the case in Germany due to the sectoral design of the health care system [20]. Therefore, the characteristics of patients presenting in our study may have contributed to the observed challenges. We did not retrospectively gather potentially relevant patient information, including socioeconomic status and health literacy data. It has been noted that patients with mild symptoms who present to an emergency department in Germany with conditions that should generally be cared for by the primary care sector tend to have a lower socioeconomic status [21]. Although data on the relationship between lower socioeconomic status and adherence are not conclusive, some studies have shown that lower socioeconomic status is associated with lower adherence, at least in certain conditions [22,23]. For future research, especially in pilot projects focused on compliance and adherence to remote monitoring programs, we suggest that it would be important to have socioeconomic status and health literacy data.

Some patients presenting to the emergency department in our study, despite only having slightly symptomatic or asymptomatic SARS-CoV-2 infection, did not use the suggested home monitoring devices at all or often stopped using them. Many of the small group of participants stopped taking home measurements around 14 days after first testing positive for SARS-CoV-2 infection; this coincides with the duration of officially mandated isolation in Germany at the time [24]. Although these recommendations were gradually eased during the course of the pandemic [25], many people were still aware of the initial regulations and were also officially advised to reduce contact for 14 days.

Another important point is that there were no immediate consequences for the patients based on whether they did or did not perform daily measurements because the data were only stored locally on their device (unless shared with a health care professional at a future appointment). Given that we have shown that there is consistently better adherence to remote COVID-19 monitoring in primary care [26], it is reasonable to assume that socioeconomic factors are not the only contributors to the low adherence to monitoring in this study. We did observe interindividual differences in the use of home monitoring, but the study population was too small to be able to make valid statements about these variations. We were also unable to determine whether the home measurements taken by study participants were useful in their medical care because the sharing of home measurements at subsequent primary care visits was only reported by one individual.

One potential conclusion based on our data is that a close physician-patient relationship and the provision of feedback are important, as documented previously [27,28]. This physician-patient relationship might not necessarily occur in the setting of emergency treatment in a large clinic with changing staff. In addition to an easily accessible contact person and adequate infrastructure, continuous support seems to play an important role. Although we educated patients about the possible benefits and insights for them personally when providing information about the study, this single contact with a physician might not provide the level of motivation that might come from a monitoring recommendation made by a more familiar health care professional, such as a general practitioner. Therefore, we suggest that the patient motivation issues seen in our study might have been mitigated by a closer physician-patient relationship or continuous feedback on device usage and readings obtained.

Our results on inclusion and device usage show that our approach, as it was, is not feasible in the German health care system without adaptations. Even if the study was planned with the existing personnel, the implementation still required additional workload and financial resources. With such a low uptake and usage rate as in our study, such an expense cannot be justified as feasible.

Concurrently, the small number of participants represents the greatest limitation of our study. Accordingly, we were unable to conduct our main objective, namely the analyses with regard to early markers of deterioration and our planned qualitative analysis in a reasonable manner. Although we are able to reconstruct the low number of participants at least in part with the explanations given above, this is not a pleasing result overall. Even if our study was able to provide important insights, it was not possible to establish a patient-led home monitoring concept.

Despite the disappointing uptake and use of home monitoring in our study, we believe that further evaluation of this approach in the tertiary care setting is warranted because home monitoring can provide valuable data for treatment and patient assessment. In specialized outpatient clinics and post-COVID follow-ups, where there is also repeated and regular contact with the same treatment team, it can be assumed that there is a stronger physician-patient relationship, which could have a positive impact on such an approach. Braun et al [29] introduced a similar concept for patients with chronic lung diseases using the same SaniQ app (Qurasoft GmbH) in routine secondary care during the COVID-19 pandemic. In this setting, patients showed satisfactory adherence and acceptance, but it must be noted that this program only required one measurement per month rather than the daily measurements in our study. Therefore, the frequency of measurements must be discussed critically when creating home monitoring concepts.

To create a reliable physician-patient relationship to maximize the benefit of home monitoring in patients with COVID-19, we advocate either the establishment of virtual wards [7,11,30] or involvement of the regular primary care sector [31], as described previously. Therefore, we suggest that the use of nonsupervised home monitoring in the primary care practice outpatient setting would be a better approach than initiation via the emergency department of a tertiary care provider.

Another factor that appears to be crucial to the success of a home monitoring program is a simple setup process. In our study, device and app setup created a lot of frustrations for both patients and the support team. Even though the setup should have been simple in principle (pairing the devices via Bluetooth with the patient’s smartphone using the app), connection errors still occurred that could not always be easily resolved. Even with telephone support from the study team, some connection problems could not be solved quickly. General problem-solving approaches did produce the desired effect, but this was very time-consuming. To avoid such frustrations at the beginning of home monitoring, we recommend the use of technology that ensures the required external devices can be connected more easily. For example, this could be achieved by means of a base station assigned to the delivered measuring devices, meaning that only the base station would need to be paired with the patient’s smartphone. Participants in our study also reported that the time burden of taking measurements was relatively high. The impact of this on motivation to continue performing daily measurements should not be underestimated. Therefore, there seems to be a need to balance the need for data density with a good user experience to create a robust and user-friendly home monitoring concept.

If we conducted the study again, in addition to the points mentioned above, we would also establish a monitoring board analogous to regulated drug trials: our analyses of the total number of potential study participants and the patients actually included were only evaluated at the end of the study and not during its implementation. If a corresponding monitoring board had been in place, the insufficient inclusion rates would have been noticed earlier so that we could have incorporated modifications into our recruitment process. A monitoring board of this kind can be operated with only a small amount of additional personnel resources if, for example, monthly intervals are selected.

Even though our aim was to investigate the potential of home monitoring with the available personnel resources, good staffing appears to be essential for the success of this kind of study: even a screening of the patient documentation by a study assistant (eg, a student assistant) and a resulting targeted indication to the study physicians could lead to potential inclusions not being missed despite the staff shortage. Once this has been established in a first phase, such support would probably no longer be necessary as soon as the staff had internalized the relevant processes.

However, such an approach would probably not have mitigated the low usage rate of the included patients. As far as can be deduced from our data and as explained above, the lack of ongoing care at home through the emergency department personnel appears to be the decisive factor here.

### Limitations

Due to staff shortages in the emergency department at the time the study was conducted, there is a risk of selection bias. For example, it may have been easier to have time to provide information about the study when the emergency department was less busy. However, the study was designed to be conducted with existing personnel resources, and therefore, it was not possible to mitigate this potential source of bias. Similarly, we are missing socioeconomic data for participants because these were not collected to avoid placing an additional workload on the medical personnel involved. However, the main limitation of our study is the small number of participants recruited, and the even smaller numbers who were active users of self-monitoring and completed the postmonitoring interview. This limits the amount of data we were able to obtain, and therefore, the conclusions that can be drawn. Consequently, we were also unable to conduct our planned analyses with regard to early markers of deterioration and our planned qualitative analysis in a reasonable manner.

### Conclusions

Due to the small sample size in this study, the main conclusion that we can draw is that providing nonsupervised home monitoring to outpatients with COVID-19 through a single contact in an emergency department of a tertiary hospital is not the optimal approach. Although conducted during the COVID-19 pandemic, our data could provide useful information about the issues related to the setup of home monitoring for a variety of diseases based on a single emergency department contact. Key features for success are likely to be regular feedback and care and an ongoing physician-patient relationship.

## Supplementary material

10.2196/58364 Multimedia Appendix 1 Interview guide.

## Abbreviations

STROBE: Strengthening the Reporting of Observational Studies in Epidemiology
