# Supplementary material for: Home Monitoring Delivered Through the Emergency Department for Outpatients With COVID-19: COVID19@Home Aachen Pilot Cohort Study
Source: JMIR Med Inform. 2025 Sep 18;13:e58364. doi: 10.2196/58364 (PMC12445781; doi:10.2196/58364)
Supplement: Multimedia Appendix 1 [file medinform-v13-e58364-s001.pdf]

COVID19@Home: Interview Guide for Assessing the Patient Perspective in the Emergency Department

Objective: How satisfied are patients with home monitoring? How useful was it?

	Topic	Questions	Notes
1	**Introduction**	**Hello, my name is ________________.**<br>**I am ________________ (describe your role at the institution).**<br>**Thank you for agreeing to this telephone interview. Is this a convenient time for you, or would another time be preferable? When would suit you best?**<br>**Today, we would like to discuss your perspective on the Covid-19@home study, in which you participated."**<br>Your perspective is very important to us, as Covid-19, or coronavirus infections, is still a relatively new disease, and we would like to understand how treatment and support for affected individuals can be optimized.	□ Introduction<br><br>□ Presentation of Covid-19@home
2	**Framework Conditions**	**"Our conversation will last approximately 20 to 30 minutes.**<br>**It is important to note that there are no right or wrong answers; we are interested in your personal opinion on this topic. You are our expert on telemedicine in the context of Covid-19, as you have used the app and devices yourself in recent weeks.**<br>**All your personal data will be anonymized so that it will not be possible to identify you afterwards.**<br>**As previously mentioned, I will record this conversation to facilitate a more accurate analysis of your responses. Is this acceptable to you?" [Start recording]**	□ Consent to recording<br><br>□ Start recording
3	**Experience with Telemonitoring**	**1. „"How did you manage with the app and devices during the Covid-19@home study?"**<br>(Could you describe your experiences with telemonitoring?)<br><br>**2. "On a typical day, how did you approach the measurements?"** (Did you perform the measurements yourself, or did someone assist you? **At what times of day did you usually take the measurements?)**<br><br>**3. "What has been your experience with conducting the measurements?"**<br>(Did the devices function reliably? Were all measurement values transmitted? Was the operation straightforward? Where did you obtain information about how to perform the measurements (GP or emergency department)?)<br><br>**4. "Did you receive any feedback from the app indicating that your measurements were in a critical range?"**<br>(How did you respond? What actions did you take? Was there anyone you reported this to when such an event occurred?)	□ Introduction<br>□ Measurement management<br>□ Usability<br>(TAM Perceived Usefulness)<br>□ Practicality<br>(TAM Ease of Use)<br>□ Usage<br>(TAM Intention to Use)<br><br>□ Compliance<br>□ Information needs<br>□ Experiences with critical values

COVID19@Home: Interview Guide for Assessing the Patient Perspective in the Emergency Department

Objective: How satisfied are patients with home monitoring? How useful was it?

		**5. "Looking back over the past few weeks, did you feel generally comfortable or uncomfortable using the app?" (positive/negative)**<br><br>**6. "Had you previously used telemonitoring applications, such as any health apps?"** (If so, which ones?)	◻ TAM Attitude towards Technology<br>◻ Experiences with telemonitoring
4	**Covid19@home**	**"Now, I would like to discuss the Covid-19@home project specifically."**<br><br>**1. "How did you come to participate in the study?"** (What motivated you to take part?)<br><br>2. **"What expectations did you have when you agreed to participate?"** (What did you hope would change for you? What did you hope would change for your GP?)<br><br>**3. "Did you share your measurement values with your GP?"**<br>**a) If yes: "How did you communicate your values (PDF, by phone, how often)?"** (How did your GP respond?)<br>**b) If no: "Is there a reason you did not share your values?"** (Did you have contact with your GP but did not mention your telemonitoring? Why?)	◻ Motivation<br><br>◻ Expectations<br>◻ Improvements<br>◻ Disappointments<br><br>◻ Communication GP-Patient<br><br>◻ Communication Patient-GP
5	**Reflection**	**1. "Looking back, what did you find positive about telemonitoring overall?"**<br><br>**2. „What did you find problematic about telemonitoring? What disadvantages do you see for yourself or for other patients using telemonitoring?"** (In your view, what might be reasons for patients to drop out of the study? Did you ever consider withdrawing from the study?)<br><br>**3. „Would you recommend the use of the app and devices to your family and friends?"**	◻ Facilitating factors<br><br>◻ Inhibiting factors<br>◻ Concerns/fears<br><br>◻ Recommendation
6	**Closing Remarks**	**"We are nearing the end of our conversation."**<br>1. **"Do you have any concrete suggestions for improvement?"** (regarding the application itself, collaboration with physicians, or support from the study team)<br>2. **"Is there anything else you would like to share? Do you have any questions, requests, or comments?"**	◻ Final questions
7	**Thanks**	**„Thank you very much for participating in this interview. Your feedback is extremely valuable in helping us to further improve the care of patients with Covid-19"**	
